# Gene Function Prediction Based on the Gene Ontology Hierarchical Structure

**DOI:** 10.1371/journal.pone.0107187

**Published:** 2014-09-05

**Authors:** Liangxi Cheng, Hongfei Lin, Yuncui Hu, Jian Wang, Zhihao Yang

**Affiliations:** 1 Department of Biomedical Engineering, Dalian University of Technology, Dalian, China; 2 School of Computer Science and Technology, Dalian University of Technology, Dalian, Liaoning, China; Harvard Medical School, United States of America

## Abstract

The information of the Gene Ontology annotation is helpful in the explanation of life science phenomena, and can provide great support for the research of the biomedical field. The use of the Gene Ontology is gradually affecting the way people store and understand bioinformatic data. To facilitate the prediction of gene functions with the aid of text mining methods and existing resources, we transform it into a multi-label top-down classification problem and develop a method that uses the hierarchical relationships in the Gene Ontology structure to relieve the quantitative imbalance of positive and negative training samples. Meanwhile the method enhances the discriminating ability of classifiers by retaining and highlighting the key training samples. Additionally, the top-down classifier based on a tree structure takes the relationship of target classes into consideration and thus solves the incompatibility between the classification results and the Gene Ontology structure. Our experiment on the Gene Ontology annotation corpus achieves an F-value performance of 50.7% (precision: 52.7% recall: 48.9%). The experimental results demonstrate that when the size of training set is small, it can be expanded via topological propagation of associated documents between the parent and child nodes in the tree structure. The top-down classification model applies to the set of texts in an ontology structure or with a hierarchical relationship.

## Introduction

One of the central purposes of genomics research is to explore the biological functions of the organism. This is exemplified by the establishment of a dynamic controlled vocabulary in the Gene Ontology (GO) database [1], which aims to interpret and annotate the role of eukaryotic genes and proteins within the cell as well as relevant biomedical knowledge, and keeps the descriptions of gene products consistent across a variety of databases. The GO annotation database contains large amounts of such function annotation knowledge, which play a key role in the interpretation of bioinformatics experiments. These annotation databases, nevertheless, are not complete. What's more, for all the organisms, we have known only a subset of all genes so far, and a much smaller subset of them are annotated with function information. The knowledge of the GO function annotation is usually extracted manually from the text data by expert staff and stored in the databases. Due to the rapid accumulation of function information in the biomedical literature, the use of text mining tools to assist with the extraction of function annotation information has become an important task. The gene function prediction methods, which annotate genes with function information automatically through the utilization of existing resources, can be roughly divided into experimental data-based methods and knowledge-based methods.

The methods based on experimental data were used widely first. They depend on first hand experimental information of genes, and usually focus on biological metrics, such as protein structure, gene sequence, protein-protein interaction, and so forth. Based on protein sequence similarity, the GO annotations of one protein can be homologously transferred to another. From the perspective of machine learning, the annotation transfer method is a nearest neighbor classifier, so we can make use of classifiers to annotate proteins, thereby determining their GO classes. In addition, we might also check the dense regions in the clustering network to verify that proteins with same labels usually occur in the same region. Lee et al. (2006) [2] proposed a Markov random field (MRF) based method that infers protein functions using protein-protein interaction data and function annotations of its protein interaction partners. They extended direct interactions to all neighboring proteins, and one function to multiple functions to understand the functions of a protein. Ko & Lee (2009) [3] used systematic feature selection methods to assess the contribution of genomic data to protein function predicting and then investigate the relationship between genomic data and protein functions. Their study used ten different genomic data sources in Mus musculus, including protein domains, protein-protein interactions, gene expressions, phenotype ontology, phylogenetic profiles, and disease data sources. In the experiment, they measured the contribution of each data set based on its prediction quality. The methods based on experimental data can only predict the functions of genes that are provided with biological measures, so they require in advance the biological measurement of the predicted genes or proteins, which is not realistic for many new entities in the text.

The methods based on knowledge rely on existing knowledge, such as the biomedical literature and GO annotation data. Literature-based methods, involving indexing, natural language processing, computational reasoning, statistical analysis, etc., usually achieve a low accuracy in gene function prediction. Done et al. (2010) [4] proposed a method of gene function annotation that adopted latent semantic indexing. From the statistics of known annotated gene databases, this method obtains the matrix of relationship between genes and labels which measures the linking intensities between them. Via the singular value decomposition of the matrix, the data are mapped to a new vector space. This operation reduces the dimension of the original vector space, and meanwhile filters the noise of the raw data. Through the analysis of new matrix elements, new annotation information is obtained, therefore accomplishing the function prediction. Verspoor et al. (2005) [5] adopted an unsupervised learning algorithm to expand the associated words of GO nodes. They thought the original associated words of a GO node were not able to indicate its correlation with the documents fully. Therefore, by expanding the associated word set of a given GO node, the indication ability is enhanced. Besides, the proteins can be represented by its context, so they regarded the function annotation of the proteins as a text classification problem. Barutcuoglu et al. (2006) [6] designed a Bayesian framework to integrate multiple classifiers conforming to the functional taxonomy constraints. The hierarchical classifiers trained on multiple data types are based on support vector machine (SVM) and their predicting results are combined in the Bayesian framework to obtain the most probable consistent set of predictions. In addition, their method is capable to implicitly calibrate the SVM margin outputs to probabilities.

**Figure 1:**
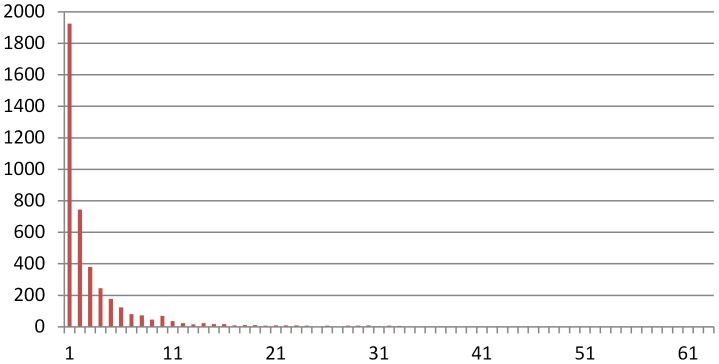
Distribution of associated document amounts of each GO term.

However, the knowledge-based methods ignore the structural features of the GO to some extent, and own the following drawbacks:

- The numbers of documents annotated by each GO term are not uniformly distributed. We investigated the distribution of associated document amounts of each GO term shown in Figure 1, where the horizontal axis is the number of documents associated with a GO term, and the vertical axis is the number of corresponding GO terms. It can be observed that many of the GO terms have fewer than 10 associated documents. It is difficult to train an accurate classifier with so few training documents. In addition, the number of samples annotated by a GO term is far fewer than that of all the rest not annotated by it, so the amount imbalance of positive and negative training samples becomes a serious obstacle to the classifier learning process.
- The output of the classifiers may be incompatible with the existing GO structure. One of the construction rules of the GO structure states that if a gene is annotated by a GO term, then it should be annotated by all of its parent items. However, the actual classification results may not always adhere to that, i.e. an instance classified as class *C* is not necessarily classified as all the parent classes of *C*.

In order to solve the above problems, we regard the gene function prediction as a multi-label classification problem, and attempt to introduce a hierarchical classification algorithm to the multi-label classification. The GO contains two types of inclusion relationship, *is_a* and *part_of*. It can be argued that these two types of relationship essentially convey certain hierarchies, so we consider that the expression of the current node can be enriched by incorporating the training samples of its ancestor node in the GO structure, which may solve the problem of insufficient positive training samples.

## Method Description

### 2.1 Multi-label Classification

In traditional classification studies, it is generally assumed that an instance corresponds only to one class label. But in reality, an instance is likely to correspond to a variety of classes. An example is that a piece of newspaper text may belong to the classes of both politics and economics. Therefore it is required that the trained classification model is capable of assigning multiple labels to an instance. A multi-label classifier is exactly such a kind of classifier.

In the problem of gene function prediction, a gene is likely to associated with multiple GO concepts. For example, the gene P25686 in the UniProt database is annotated by GO terms with *id* 0032436 (positive regulation of proteasomal the ubiquitin-dependent protein, catabolic process), 0090086 (negative regulation of protein deubiquitination), 0030433 (ER-associated protein catabolic process), 0031398 (positive regulation of protein ubiquitination), 0090084 (negative regulation of the inclusion of the body assembly). These GO concepts together describe the gene functions: protease-based pan-hormone catabolic process positive regulation of protein de-ubiquitin negative adjustment, the ER-associated protein catabolic process, positive regulation of protein ubiquitin, the virus endosome assembly negative regulation. Therefore, we may regard the prediction of gene function as a problem of multi-label annotation, namely selecting several GO concepts as function description of a given gene. Due to the descriptive precision and free obtainment of biomedical abstracts, this paper focuses on the abstract-level classification, namely determining which GO terms a given biomedical abstract is associated with.

Multi-label classification methods can be grouped generally into two types: the methods based on problem transformation and the methods based on algorithm modification. The basic idea of the methods based on problem transformation is to transform the multi-label classification problem into multiple single-label classifications, so that existing single-label classification methods can be used to settle the multi-label classification problems. The prediction of each class is treated as an independent single-label classification, which owns an individual classifier. All of the training data are used to train each classifier, therefore constructing a classification model applicable to that class. Due to the assumption that all classes are independent of each other, the disadvantage of such methods is the neglect of the relationships among these classes. Furthermore, the training of each classification model needs to exploit all the training data, which often leads to an amount imbalance of positive and negative training samples and thereby has an adverse impact upon the classification. The principle of the methods based on algorithm modification is to modify the existing single-label classification algorithm so as to be capable of handling multi-label classification problems. Rank-SVM (Elisseeff & Weston, 2003) [7] is a modification of the basic SVM algorithm. It develops a sorting approach to select a subset of the whole classes in the class prediction of a given sample, which in fact converts the multi-label prediction to a quadratic programming problem. The ML-kNN algorithm (Zhang & Zhou, 2005) [8] is a transformation of the k-nearest neighbor algorithm. This method acquires the a priori probabilities of each class through a statistical method, and then for each sample calculates the posterior probabilities according to Bayes' rule, in order to determine the sample classes.

Given a gene and its associated literature, we do annotations according to the classification result of the literature in this paper. Here the classifiers are trained based on supervised learning, with the words of the texts as the input features and the GO terms as the target classes. To determine the classes of the genes, we take the problem transformation based methods, and train classifiers for each GO term. When predicting the classes of a given gene, all the classifiers are called and finally those classes whose classifiers return positive value are the labels of that gene.

### 2.2 The Top-down Multi-label Annotation

The hierarchical classification method generally refers to a method that organizes all the classes into a tree structure according to a certain hierarchical relationship, and then with the idea of “Divide-and-Conquer”, assigns the instances to be classified to the nodes in the tree. This type of classification method is more accurate than ordinary classification methods. Common methods used for hierarchical text classification include the flat classification and the top-down classification. In the flat classification, the hierarchical relationship in the tree structure is “flattened”. The construction of a classification model for a node does not consider other nodes; in other words, this method ignores the association information in the tree structure of classes. Of all the training samples, there are some representative ones expressing subtle differences among the classes, which we believe may provide essential instruction for the classification. During the training process of the flat classification model, those samples are mixed with large numbers of other training samples and submerged, so the classifiers are not able to classify effectively these samples at the class boundaries. In this paper, by selecting the training samples properly for each node in the tree structure, the classifying ability is enhanced, and thereby it achieves a more accurate classification result.

In the present study, the gene annotating is transformed into a classification based on the GO structure. We build a GO graph using the biological process branch data of Gene Ontology Annotation (GOA) resources. In this graph, the nodes represent GO terms and the edges indicate semantic relationship between the GO terms. The graph structure conforms to the definition of the biological process branch given by the GO annotation institutions. Further, the information within the graph is enriched by adding the associated PubMed literature to GO nodes, which makes it feasible to construct a text classifier for each node. In our methods, each node corresponds to two PubMed identifier (PMID) sets. The first set contains the identifiers of PubMed documents directly associated with the current node, namely the identifier set of PubMed documents annotated by the current GO term, which we call “the current node associated document set” (denoted by *CurNodePMIDSet*); the second set is obtained via topological propagation across the entire GO tree structure, so it contains the identifiers of those PubMed documents associated with the current node or its descendant nodes, which we call “the descendant node associated document set” (denoted by *DescNodePMIDSet*). Although this study focuses on the annotation of biological process branch of the GO, the method also applies to the other two branches of the GO, molecular function and cellular composition.

Because of the sparsity of the associated PubMed documents for a given GO node, we try to expand its positive sample set and reduce the negative sample set during the training set construction, thereby relieving the amount imbalance of positive and negative samples. For a GO node, the documents, which are associated with it or its descendant nodes (namely the documents in *DescNodePMIDSet*), are annotated as positive training documents. As opposed to simply using the documents associated with the current GO node (namely the documents in *CurNodePMIDSet*), it enriches the information about positive samples. On determining the set of negative training samples, we focus primarily on the differences between parent and child nodes in the GO structure, in other words, those samples which are associated with the parent nodes of the current node but are not inherited by the current node or its child nodes are selected as negative samples. We believe that such samples are able to well represent the differences between parent and child nodes, and do good to classifying the samples located at the class boundaries. Consequently, the set of negative documents is the remainder of the union of all the *DescNodePMIDSet*s corresponding to the current's parent nodes minus the *DescNodePMIDSet* of the current node. Such an adjustment of negative document set retains the key samples between parent and child classes, as well as greatly reduces the size of negative sample set.

Both the flat and top-down classification methods were applied to the gene function prediction task in this paper, and then the results were compared. In the flat classification method, for each node in the GO structure, the documents in its *CurNodePMIDSet* are regarded as positive training samples, while all the other documents in training set are regarded as negative ones. The top-down classification method takes into account the relationship between target classes in the training process, so the multiple output labels are not compatible with such relationship. At the training stage, the classification model is constructed respectively for each node in the GO structure. Then at the predicting stage, for each document to be classified, starting from the root node, the classifier of the current node is applied to determine whether the given document belongs to the current class. If it does, then the classifiers of its child nodes continue classifying the given document further, and this process is carried on until reaching the leaf nodes; otherwise the classification process stops. In this way it is guaranteed that a document belonging to a certain class is destined to belong to its parent classes, or the classification process is impossible to reach the node of this class. Such a top-down classification process effectively settles the problem mentioned above that the classification results are not compatible with the GO structure.

### 2.3 Detailed Procedure

The detailed procedure of corpus processing and further generating a classification model for each GO node is as follows:

Step 1. Relation extraction: extract *is_a* and *part_of* relations from the biological process branch of the GO to obtain the hierarchical relationship between every pair of parent and child nodes, and determine the child node set and the descendant node set of each node. Finally a directed graph of GO terms is constructed.
Step 2. Association resolution: resolve the association file of genes, GO terms and PubMed documents, and then obtain a set of the current node associated PMIDs for each GO term (namely *CurNodePMIDSet*).
Step 3. Topological propagation: based on the child nodes set acquired in Step 1 and the current node PMID set acquired in Step 2, topologically sort the whole graph. PMID sets associated with nodes are propagated from child nodes to parent nodes, and the descendant node associated PMID set (namely *DescNodePMIDSet*) is generated for each node.
Step 4. Abstract extraction: extract abstracts of the PMID mentioned in any *CurNodePMIDSet* or *DescNodePMIDSet* from the whole MEDLINE abstract documents. Stop words are removed from the extracted abstracts and then a vector space model is built for each processed abstract so that it can be represented with a vector.
Step 5. Classification model construction: traverse each GO node of the directed graph and construct for each node two classification models based on naive Bayes and SVM respectively. Finally the 10-fold cross-validation is adopted: each time one tenth of abstract documents are selected as the test set, with the rest as the training set.

### 2.4 Experimental Settings

The whole abstract documents were divided into 10 parts, and according to the 10-fold cross-validation method, every part was selected as a test set in turn and the rest used for training. The present paper checked the effects of the flat classification and top-down classification method respectively, and furthermore made a comparison of their classification results. The experimental settings are as follows:

- The flat classification: for a node in the GO structure, the documents associated with the current node, namely belonging to the *CurNodePMIDSet*, are regarded as positive training samples, while all other documents of training set as negative training samples.
- The top-down classification: for a node in the GO structure, the documents associated with the current node or its descendants, namely belonging to the *DescNodePMIDSet*, are regarded as positive training samples, while the union of *DescNodePMIDSet* of all parent nodes minus the *DescNodePMIDSet* of the current GO node gets the negative training sample set.
- Implementation: the concrete classification algorithms are implemented with naive Bayes and SVM, respectively.

## Experimental Results and Analysis

### 3.1 The Experimental Data

**Figure 2:**
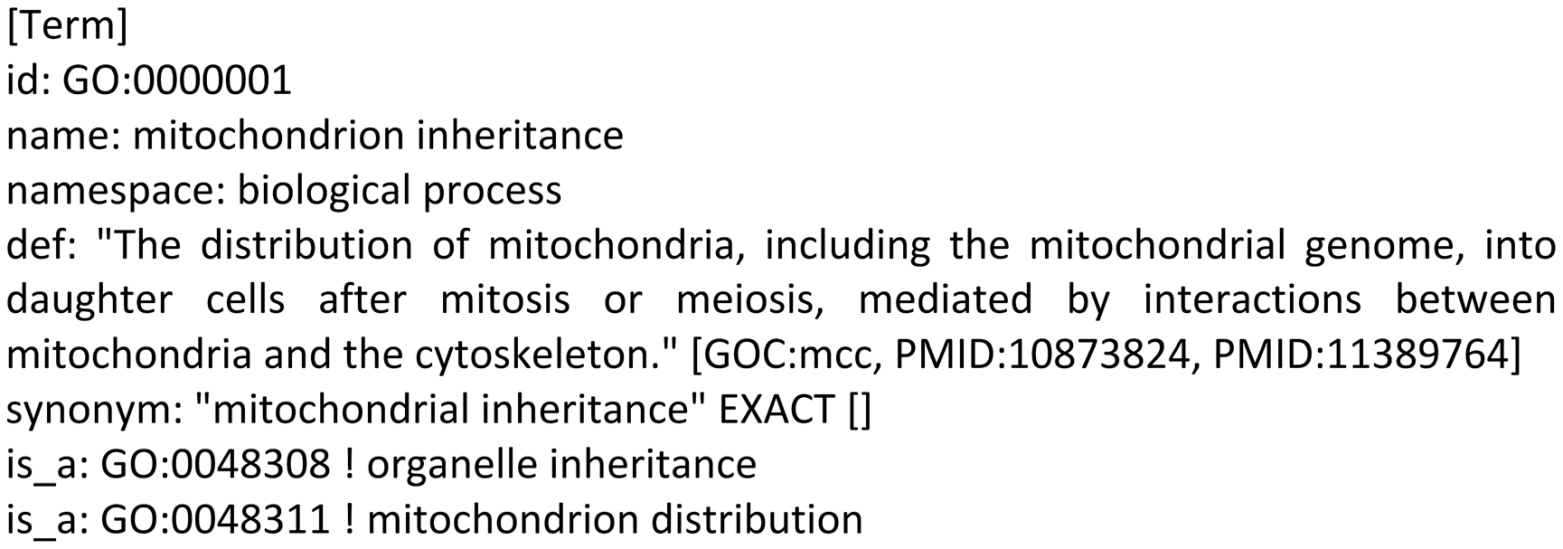
The file format of the Gene Ontology.

a. The Gene Ontology files. We downloaded the ontology files (2010 version) from the GO website [1], and extracted all the items of the biological process branch, a total of 20385 terms with the format shown in Figure 2.

In Figure 2, the *id* entry shows that the identifier of this GO term in the entire ontology structure is GO:0000001, and the *name* entry shows that this term represents mitochondrion inheritance. The *namespace* entry shows that this term belongs to the biological process branch, which is one of the three sub-hierarchical structures of the GO hierarchy. Two *is_a* entries indicate that the term has two parent nodes, namely organelle inheritance with *id* GO:0048308 and mitochondrion distribution with *id* GO:0048311. By resolving all the GO data, we find that the child node of term GO:0000001 is the term mitochondrial DNA inheritance with *id* GO:0033955.

b. The Gene Ontology association file. Download from the GO website [1], this file records concretely the relationships between each gene in the UniProt database and each GO term. The records are in the form of a triplet <gene, GOID, PMID>. The entries of this triplet represent respectively, from left to right, the identifier of the gene in the UniProt database, the identifier of GO term associated with the gene and the PMID of the evident data confirming reasonability of this annotation. There are a total of 198021 such records in this file.
c. The MEDLINE abstract file. Downloaded from the U.S. National Center for Biotechnology Information (NCBI) website [9], this file contains the title and abstract contents of MEDLINE documents prior to 2008.

### 3.2 Evaluation Methods

In this paper, we adopt the precision, recall and F-value to evaluate the methods in this paper. Because the gene function annotation methods in the present paper construct a classifier for each node in the GO structure, the measures of precision and recall are obtained by summing the results of all classifiers. The evaluation metrics are defined as bellow, in which *Y_i_* stands for the prediction of the classifier corresponding to node *i* in the GO structure, and *Z_i_* stands for the standard answer.
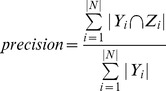
(1)

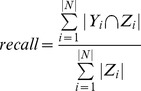
(2)

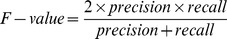
(3)


### 3.3 The Experimental Results

**Figure 3:**
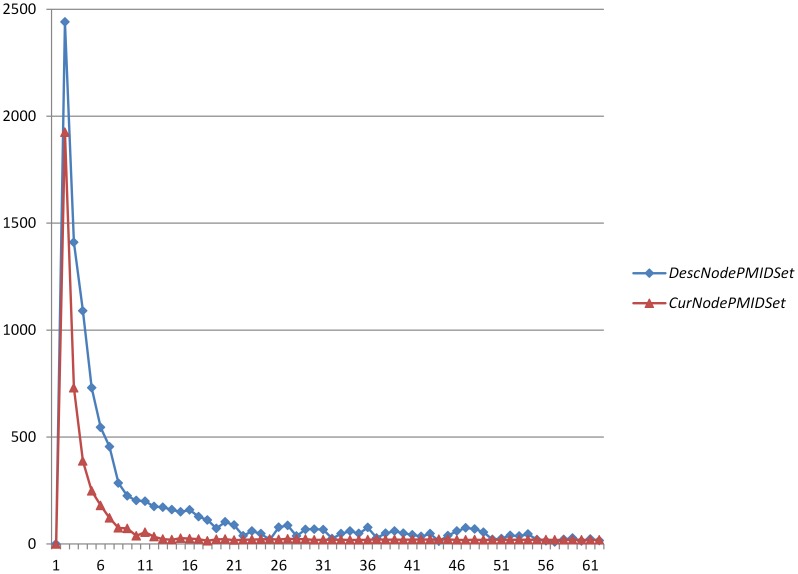
Distribution of the document numbers in *DescNodePMIDSet*/*CurNodePMIDSet* of each GO node in the tree structure.

Table 1 contrasts the numbers of positive and negative training samples for the flat classification and top-down classification, which are acquired by calculating the average numbers for all GO nodes. Figure 3 shows the distribution of the document numbers in *DescNodePMIDSet*/*CurNodePMIDSet* of each GO node in the tree structure, where the horizontal axis is the document numbers in *DescNodePMIDSet*/*CurNodePMIDSet*, and the vertical axis is the number of GO terms having the corresponding number of documents in *DescNodePMIDSet*/*CurNodePMIDSet*. The curve corresponding to *DescNodePMIDSet* is almost above the one corresponding to *CurNodePMIDSet*. It clarifies that via topological propagation between parent and child nodes in the tree structure, the additions of associated documents for GO nodes lead to a general increase in the number of positive training samples, while the parent-child node differences are taken into account to narrow the size of negative training sample set additionally. Because of the great similarity between the parent and child nodes, we selected the most difference-distinguishing samples between them as negative samples, which solves the imbalance of negative and positive training samples to some degree.

**Table 1:** The Comparison of Average Numbers of Positive and Negative Training Samples.

	Average Number of Positive Training Samples	Average Number of Negative Training Samples
the Flat Classification	2.136	10827
the Top-down Classification	3.864	996

**Figure 4:**
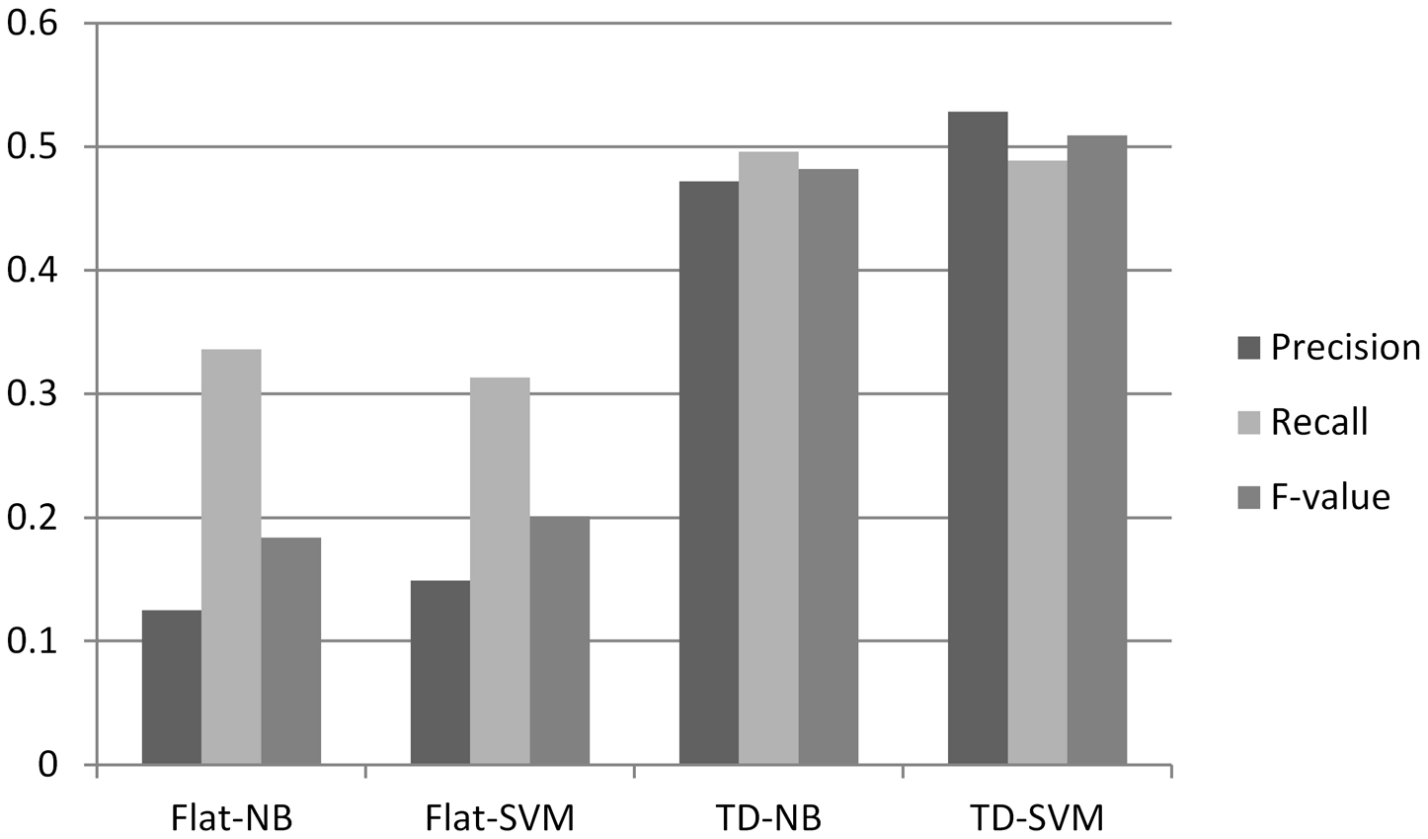
Performance of different experimental settings.

The results of gene function prediction are shown in Figure 4, where the Flat-NB represents the flat classification result that is implemented by a naïve Bayes classification model, the Flat-SVM represents the result of the flat classification based on SVM, and the TD-NB and TD-SVM represent the top-down classification result based on naive Bayes and SVM, respectively. It shows the results of classification based on SVM are slightly better than that based on naive Bayes. The SVM classification model is based on geometric principles and each text term is an attribute of the text. These multiple attribute dimensions form a vector space and a text can be represented by a point in that space. Then the purpose of a SVM classifier is to determine a hyper plane that is able to separate the positive and negative samples correctly. Compared to the naive Bayes' classifier based on statistical probabilities, the SVM model has richer representations for documents, thereby resulting in the better performance observed in the experimental results. The latter two groups of results, which are produced by the top-down classification methods, improve significantly in contrast with the first two groups by the flat classification methods. The construction of training samples based on the hierarchical relationship among the GO nodes, not only reduces the size gap between the positive and negative samples, but enhances the instructional role of negative training sample set, and therefore generates a more accurate classification model.

From the comparison of experimental results, we can see that the top-down classification model excels over the flat classification model when the samples are in a hierarchical relationship. The results show that when the training set is small, our method is able to increase the number of positive samples via topologically propagation between parent and child nodes, and constructs a training sample set with greater difference-distinguishing ability. The best results are obtained in the top-down classification based on SVM, where the precision, recall and F-value are 52.7%, 48.9% and 50.7% respectively. Barutcuoglu et al. (2006) [6] examined predictions using old snapshot data before and after Bayesian correction. They achieved 32% precision and 7% sensitivity (recall) with independent SVM classifiers, and the sensitivity was increased three times (21%) at comparable precision (31%) after adopting Bayesian correction. By taking the hierarchical structure of functional classes into account, their results were significantly improved. Compared with the results of Barutcuoglu et al., the best results of our method excels, demonstrating the effectiveness of the top-down classification based on SVM. This is probably because that taking into consideration the tree structure of GO nodes, we not only build a top-down classification model, but also adjust the training samples and expand the positive training sample sets, reducing the imbalance between the positive and negative samples, both of which jointly lead to a better gene function predicting result.

## Conclusions

For the annotations of the gene functions, which can be interpreted to explain the phenomena of life science, this paper studies how to use text mining methods to assist gene function prediction, and further transforms it into a multi-label hierarchical classification problem. By making use of the hierarchical relationship of the GO structure to adjust the training samples, our method expands the positive training sample sets and reduces the imbalance between the positive and negative samples. Meanwhile, we retain and highlight most distinguishing training samples to enhance the classification ability. The top-down classifier, constructed from the tree structure, can solve the incompatibility between the classification results and the GO structure in that it takes into consideration the relationship between target classes during the training and predicting process. Such a top-down classification model applies to a set of texts in a hierarchical relationship.

There is a drawback with the gene function prediction method used in this paper. If a classifier of internal nodes produces an error in the classification process, this error will propagate downward to leaf nodes. We need to minimize the error propagation between nodes with an inheritance relation to improve the accuracy of classification. Some further approaches could be adopted to correct the classification results, such as the multi-classifier merging.
